# Genetic Engineering of Resident Bacteria in the Gut Microbiome

**DOI:** 10.1128/jb.00127-23

**Published:** 2023-06-29

**Authors:** Jack Arnold, Joshua Glazier, Mark Mimee

**Affiliations:** a Pritzker School of Molecular Engineering, University of Chicago, Chicago, Illinois, USA; b Department of Microbiology, University of Chicago, Chicago, Illinois, USA; University of Chicago

**Keywords:** microbiome, synthetic biology, nonmodel organisms, CRISPR-Cas, metabolic engineering

## Abstract

Techniques by which to genetically manipulate members of the microbiota enable both the evaluation of host-microbe interactions and an avenue by which to monitor and modulate human physiology. Genetic engineering applications have traditionally focused on model gut residents, such as Escherichia coli and lactic acid bacteria. However, emerging efforts by which to develop synthetic biology toolsets for “nonmodel” resident gut microbes could provide an improved foundation for microbiome engineering. As genome engineering tools come online, so too have novel applications for engineered gut microbes. Engineered resident gut bacteria facilitate investigations of the roles of microbes and their metabolites on host health and allow for potential live microbial biotherapeutics. Due to the rapid pace of discovery in this burgeoning field, this minireview highlights advancements in the genetic engineering of all resident gut microbes.

## INTRODUCTION

**FIG 1:**
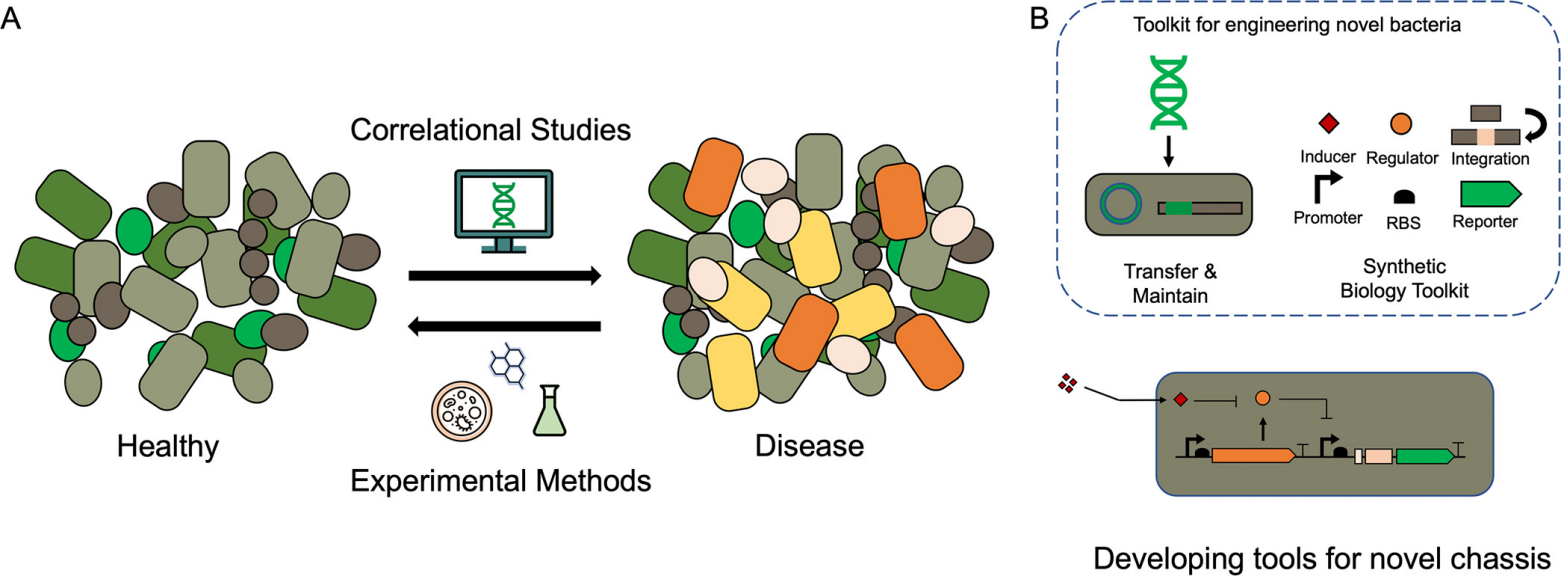
Increasing microbiome knowledge enables the engineering of novel bacterial species. An increased number of (A) correlational studies have linked shifts from a healthy gut to various disease states to corresponding changes in the gut microbiota. Recently developed experimental and predictive methods that explore this correlational information can be used to understand factors pertaining to healthy microbiota states and to generate (B) genetic tools with which novel bacterial chassis in the microbiome can be tamed. These tools include, but are not limited to, plasmids and genome integration strategies for the transfer and maintenance of genetic material as well as parts from synthetic biology toolkits to control gene expression, such as promoters and ribosome binding sites (RBS).

The human gastrointestinal (GI) tract is an important organ system at the nexus of host metabolism, immune system regulation, and host-microbe interactions. The gut is home to trillions of microorganisms that interact with each other and the host to shape this dynamic environment. Many of these microbes are implicated in human health and development, and the alteration of microbial populations is strongly correlated with numerous disease states (Fig. 1A) (1–5). Microbes in the human gut offer a unique access point to indirectly monitor or modulate the host physiological processes. The genetic engineering of gut microbes provides opportunities to probe the underlying molecular mechanisms of host-microbe interactions in the GI tract and to indirectly modulate host function. These investigations can both enhance our basic understanding of the microbiome and lead to the development of novel microbiome-based therapeutic and diagnostic systems.

## WHY ENGINEER RESIDENT GUT MICROBES?

Traditionally, methods by which to engineer bacteria from the microbiome have been limited to a few well-studied, readily culturable bacteria. Using tools and strategies from synthetic biology, microbiome engineering has focused on these “model” chassis organisms, such as Escherichia coli and Lactococcous lactis. For example, E. coli has been used as a diagnostic to detect and treat inflammation in mice (6–9) as well as gastric bleeding in pigs (10) and as a live biotherapeutic of phenylketonuria (PKU) in mice and primates (11). Seminal work in engineering L. lactis to secrete IL-10 has also been shown to ameliorate colitis in mice (12). While these model microbes are well-suited for some microbiome engineering applications, other less-studied gut microbes offer unique advantages:

### Direct mechanistic studies.

Profiling the microbiome in disease states sheds light on the roles microbes might play in disease progression. The molecular mechanisms that drive these microbial shifts and commensurate host responses can be probed through gain-of-function and loss-of-function studies using engineered microbes (13–16). Genetic manipulations allow for deeper mechanistic insight beyond species-phenotype relationships and facilitate the assignment of function to genes in poorly studied organisms.

### Colonization advantages.

The introduction of exogenous microbes into an established microbial community remains a significant obstacle to microbiome engineering applications. Native microbiome members may overcome colonization resistance, as they are innately well-adapted to the target environment (17). By using microbes that stably engraft in a microbiome, engineered live biotherapeutic systems can reside in the target environment for the long-term without the need for regular dosing, allowing for the autonomous control of environmental conditions.

### Intrinsic metabolic properties.

Diverse members of the gut microbiome produce unique compounds that are thought to alter the intestinal environment and affect host function, such as short-chain fatty acids (SCFAs), secondary bile acids, and antimicrobial peptides, among other potentially beneficial metabolites (15, 18, 19). By engineering resident gut microbes, we can tap into the natural metabolic diversity of gut bacteria without needing to completely rewire the metabolism of a chassis organism.

Despite these intrinsic advantages, key limitations currently the impede universal adoption of resident gut bacteria as chassis for microbiome engineering applications. Chiefly, most genetic engineering strategies rely on empirical knowledge to introduce, maintain, and manipulate recombinant DNA in a target organism. The challenges of culturing many members of the human microbiome have limited our understanding regarding the genetic composition and metabolic potential of individual species. As a result, the development of genetic tools and engineering strategies has been slow until recent years. New culture techniques, improved microbial ’omics strategies, and increased interest in the gut microbiome has spurred investigations into the physiology of gut microbes, revitalizing engineering attempts (Fig. 1A). This minireview details recent advancements in tools and techniques used to engineer resident gut bacteria and how the application of these strategies will help answer fundamental questions in microbiome research and develop novel therapeutics.

## SYNTHETIC BIOLOGY STRATEGIES FOR ENGINEERING RESIDENT GUT BACTERIA

A main objective of synthetic biology is to predict and control the behavior of a bacterial population in diverse environments. Gene expression and cellular function are programmed by using a toolset of regulatory genetic elements, including promoters, ribo-regulation systems, and CRISPR/Cas-based regulation systems in concert with functional genes (Fig. 1B) (20–25). With the existing toolsets available for model organisms, such as E. coli or L. lactis, synthetic circuits can be designed to implement diagnostic biosensors or to create cell-based factories for therapeutic immunomodulatory compounds (10, 12, 26, 27). However, these genetic parts and associated design rules are idiosyncratic to the chassis organism. For most resident gut bacteria, new pipelines for tool development are needed (16, 23, 28, 29). As many of these workflows rely on genomic and transcriptional analyses, curating high-quality data for these gut species is a critical first step to implement these strategies.

## MICROBIAL ’OMICS ADVANCEMENTS ENABLE THE GENETIC ENGINEERING OF RESIDENT GUT ORGANISMS

Traditional methods by which to analyze the taxonomic composition of a microbial community using culturing techniques have largely been replaced by ‘omics profiling, broadly referring to metagenomics, metabolomics, transcriptomics, and proteomics. With ‘omics advancements, researchers can now rapidly assess the composition of complex environmental samples without the need to culture individual species (30–32). These systems-level techniques have enabled the assembly of high-quality genomes, the prediction of unique metabolomic signatures within a given community, and the generation of compelling hypotheses of microbe-microbe and microbe-host interactions (33, 34). Given this wealth of top-down information, the main bottleneck in probing host-microbe interactions is the challenge in performing gain-of-function and loss-of-function studies *in situ*. The efficient and reliable editing of resident gut microbes can allow for these necessary genotype-to-phenotype studies to mechanistically link microbiome functions to host responses.

## HARNESSING NOVEL BACTERIAL CHASSIS FROM THE GUT MICROBIOME

### Introducing synthetic DNA.

The first step in cellular engineering is introducing new genetic material into the cell. Although many gene transfer methods exist, three methods are most often employed to edit resident gut bacteria: transformation relying on natural competence, electroporation, and conjugation from a donor organism (35, 36). A subset of bacterial species is naturally competent, meaning they can uptake foreign DNA directly from the environment (37). However, a few of these species are naturally competent in a laboratory setting, precluding widespread application of this method to introduce synthetic genetic material (38). Electroporation, another method, relies on an electric pulse to facilitate the transport of foreign DNA across the bacterial membrane. Electroporation can be parallelized to determine the appropriate conditions for poorly described gut bacteria in a high-throughput manner, but it is challenging to optimize, especially when working with oxygen-sensitive members of the gut microbiota (39). Similarly, chemically competent transformation permeabilizes bacterial membranes to allow DNA transfer. While widely used with E. coli, this strategy is infrequently used with other bacterial species and can be challenging to implement with resident gut bacteria, especially obligate anaerobic species (40, 41). Lastly, the transfer of genetic material can be mediated through conjugation by a donor strain, such as E. coli, to a resident gut recipient. While perhaps the most widely successful across various taxa, E. coli, can be toxic to some recipient species, selection and counterselection schemes can be challenging to identify, and mismatches in epigenetic modification between donors and recipients may impede the uptake of transferred genetic material (42–46). Altogether, while several techniques are available to introduce new genetic material into the cell, empirical testing is required to develop streamlined protocols for genetic manipulation.

Once in the cell, exogenous DNA must bypass bacterial defense mechanisms against foreign DNA for successful maintenance in the host. Many bacterial species encode restriction nucleases to digest foreign genetic material during DNA uptake. Several strategies to overcome these restriction barriers have been employed in recent years. For example, Zhang et al. developed E. coli strains to mimic known DNA methylation machinery by expressing heterologous methyl transferases from the *Bacillius* species, which they attempted to engineer (47). A restriction-modification (RM) system database, known as REBASE, was developed to catalog the RM systems and their associated machinery from fully sequenced genomes to aid in this technique. The ever-expanding REBASE coupled with an E. coli strain harboring the appropriate methyltransferases can ensure that cargo DNA is correctly methylated to avoid endogenous RM systems in many resident gut bacteria (48). Another mechanism to overcome the RM barrier is to systematically remove host RM recognition sites, thus creating “RM-silent” cargo DNA not targeted for deletion by host defense systems (49).

### Plasmid-based tools.

After evading native defense mechanisms, genetic cargo must be maintained and replicated along with the host chromosome. Commonly, plasmids, circular, independently replicating, and extrachromosomal DNA are used to introduce synthetic DNA and control gene expression since they are readily maintained under selection and can easily be edited (46, 50). Plasmid backbones contain a compatible replication origin to replicate in the desired chassis organism and a compatible E. coli replication origin to facilitate cloning and plasmid construction (28, 44, 46). Previously, broad host range plasmids had success in transferring genetic material to model intestinal bacteria, including E. coli and lactic acid bacteria (LAB) (45). However, work from Jin et al. has introduced a high-throughput technique to test the compatibility of different replication origins in resident gut species (16). This strategy capitalizes on the recent success of Sheridan et al. by utilizing modular Clostridia shuttle plasmids to introduce synthetic DNA to previously intractable members of the gut microbiome (43, 44). In the method introduced by Jin et al., mixed conjugation is used to test multiple replication origins at once for streamlined testing (16). Through these advancements, large numbers of resident gut bacteria can be pooled for screening, vastly expediting the time to tame and edit resident gut species.

Plasmid-based systems must also contain selective markers for maintenance within the host. Selection is typically achieved with antibiotic resistance genes or auxotrophic markers so only cells that contain the plasmid survive in growth media supplemented with or deficient in a specific compound, respectively. However, these methods can be challenging to implement in poorly studied resident gut species (28). Predictive tools have been developed to discover potential antibiotic resistance genes compatible with a target organism so that novel selectable markers can be readily identified (51). Additionally, a recent tool developed to analyze amino acid biosynthesis pathways can be used to locate potential auxotrophic markers in target hosts (52). These methods can facilitate the discovery of an appropriate method by which to select for synthetically introduced genetic material to diverse resident gut bacteria.

### Genome engineering strategies.

Although plasmids are an effective tool, their inherent instability, variable copy numbers, incompatible replication origins, and needs for continuous selection pressure are not ideal for some applications (46, 53). Integration-based systems can bypass many of these limitations by inserting cargo directly into the host genome for stable expression and replication. Classic double crossover homologous recombination techniques can be used to delete or integrate genes into the bacterial chromosome. This strategy pairs an antibiotic resistance marker that selects for the integration of a suicide plasmid into the bacterial chromosome with a counterselection marker, such as a genetic kill-switch (54, 55) or conditionally lethal gene (56) to excise the plasmid backbone to allow for a markerless deletion. Although this strategy is flexible with microbes that are not well characterized, the process is lengthy and depends on both on a reliable counterselection method and recombinogenic chassis organism.

Alternatively, integrase-based systems can streamline the insertion of genetic payloads into the bacterial chromosome. Integrases are enzymes that catalyze site-specific recombination between two DNA elements such that incoming DNA is inserted at a target genomic location. Notably, the NBU1 and NBU2 integrase systems have been widely used to engineer members of the *Bacteroides* genus (23, 54, 57, 58). With the description of thousands of predicted integrases in diverse clades of bacteria (59), future work could expand the use of integrases in resident gut systems other than *Bacteroides*.

Similar to integrase systems, integration can be mediated through the group II intron system, which uses the Ll.LtrB group II intron from L. lactis. LtrA, an intron-encoded protein, mediates self-insertion into a defined, reprogrammable target site in the host’s genome (60). This process successfully engineered the model E. coli species and is the basis for the ClosTron system used for engineering many *Clostridium* species, a genus of bacteria found in many environments, including the human gut microbiome (61, 62). When targeted to a partial 16S rRNA consensus sequence, the group II intron system can be used as an integration system (16). As a viable transfer method to many resident gut organisms, group II introns can be tailored further for synthetic biology applications. Minor modifications can be added to the intron sequence that does not impede folding and allow for the delivery of payloads, such as phage attachment (*attP*) sites. This strategy has been used to introduce *lox* sites to model and resident gut hosts where the intron containing the *lox* site acts as a “landing-pad” for a cargo vector containing complementary *lox* sites. Integration of the genetic cargo can then be incorporated into the target genome by expressing the *Cre* recombinase (62, 63). *Lox*-based landing-pad sites have also successfully transferred genetic material to resident gut hosts when coupled with transposases, such as in the CRAGE system (64).

CRISPR-Cas systems can also facilitate the integration of genetic material into bacterial genomes (27, 65–67). The CRIPSR-Cas system is an RNA-guided endonuclease that can induce double-stranded breaks in target DNA sequences in a homology-directed manner. In general, targeted genomic edits are made via homologous recombination, and CRISPR-Cas is programmed to introduce double-stranded breaks in the wild-type locus both to increase the recombination rate and for counterselection against unedited genomes. Many resident gut microbes, such as Firmicutes, one of the largest represented phyla in the human microbiota, have poorly functioning homology-directed repair (HDR) mechanisms (15). Various CRISPR systems must be screened to optimize editing efficiency in resident gut bacteria with inefficient HDR, such as pathogenic Clostridioides difficile (68, 69), to address this limitation. Tuning Cas protein production can also improve genome editing efficiency by decreasing the burden of endonuclease activity. The RiboCas system employed by Cañadas et al. uses a theophylline riboswitch to selectively induce Cas9 production for the duration of the allelic exchange reaction (70). While CRISPR systems can still be lethal to many species of gut bacteria, these advancements in decreasing lethality and alleviating cellular burden have significantly derisked these tools as viable genome editing systems. Further, newer CRISPR-transposon systems promise direct, programmable integration of DNA payloads into target loci (71). Although these systems have only been prototyped in Proteobacteria, they could be adapted to facilitate engineering members of other phyla in the gut microbiota.

## TOOLS TO REGULATE GENE EXPRESSION IN NOVEL BACTERIAL CHASSIS

### Transcriptional control of gene expression.

Engineering promoters to control gene expression are key tools in the synthetic biology toolbox and the fundamental entry point for developing more complex genetic circuitry. In well-studied gut bacteria such as *Bacteroides*, libraries of constitutive and inducible promoter systems have been rigorously tested and optimized to enable fine-tuned control of endogenous and synthetic genetic systems (22, 23, 72–76). For example, after investigating natural promoter systems, Whitaker et al. identified strong promoters in *Bacteroides* species and decoded their conserved motifs for tunable promoter engineering (76). Inducible systems have also been adapted from model bacteria and used in well-studied resident gut organisms to investigate a wide array of hypotheses *in situ*, such as how gut bacteria can alter the composition of the intestinal mucosal layer (22). In other less studied microbes, such as C. difficile, inducible saccharide utilization operons, such as the pXyl (xylose-responsive) system, have similarly been developed (77). However, in many resident gut bacteria, the challenges of *in vitro* culturing and reliable genetic editing techniques have made traditional testing and optimization of these tools difficult. Advancements in computationally guided techniques have helped mitigate these difficulties by discovering promoter signatures based on metagenomic data. Wilson et al. used large-scale RNA-Seq data sets to characterize native promoters and identify their motifs, enabling a fundamental understanding of promoter function in poorly characterized organisms and forming a basis for future engineering applications (78). Similar tools have facilitated the discovery of other transcriptional regulatory elements, such as inducible systems and transcriptional terminators, in diverse bacterial hosts (29, 79). In addition, the RBS Calculator, a computational strategy that is based on the analysis of ribosome binding sites (RBS), can be used to predict protein translation rates and design novel RBS that control the rate of translation in a chassis organism (80, 81).

### Post-transcriptional control of gene expression.

RNA and protein-based systems can similarly be used to control gene expression. A prime example of RNA-based regulation includes the use of riboswitches, which are RNA hairpins in the 5’UTR of transcripts that respond to specific ligands. Natively found in many species of bacteria, riboswitches allow for rapid and tuned responses to changing environmental stimuli and have wide-ranging applications, from gene expression modulators to sensor systems for diagnostic applications (70, 82, 83). Programmable regulation utilizing CRISPR systems can modulate gene expression at the protein level. Recently, both deadened Cas9 and Cpf1 systems have successfully been used to restrict gene expression in resident gut bacteria (16, 72). This CRISPR interference (CRISPRi) system is a particularly attractive strategy for gene regulation, as it uses small programmable gRNAs with the deadened Cas9 or Cpf1 to repress targeted endogenous genes and allows for the probing of sequence-to-phenotype relationships.

The high-throughput creation of these individual components of molecular toolsets lays the groundwork for the building of comprehensive toolkits with which to transition resident gut bacteria into new chassis organisms. These studies outline a blueprint for introducing synthetic genetic material to resident gut hosts, optimizing gene expression through promoter and RBS engineering, and characterizing inducible transcriptional and posttranscriptional regulatory systems to control gene expression. By developing empirical knowledge of these systems, individual techniques can be combined to introduce, maintain, and manipulate recombinant DNA in resident gut systems and facilitate novel hypothesis testing in the gut.

## APPLICATIONS OF ENGINEERED RESIDENT GUT BACTERIA

### Uncovering microbe-host interactions and the roles of microbially derived metabolites.

**FIG 2:**
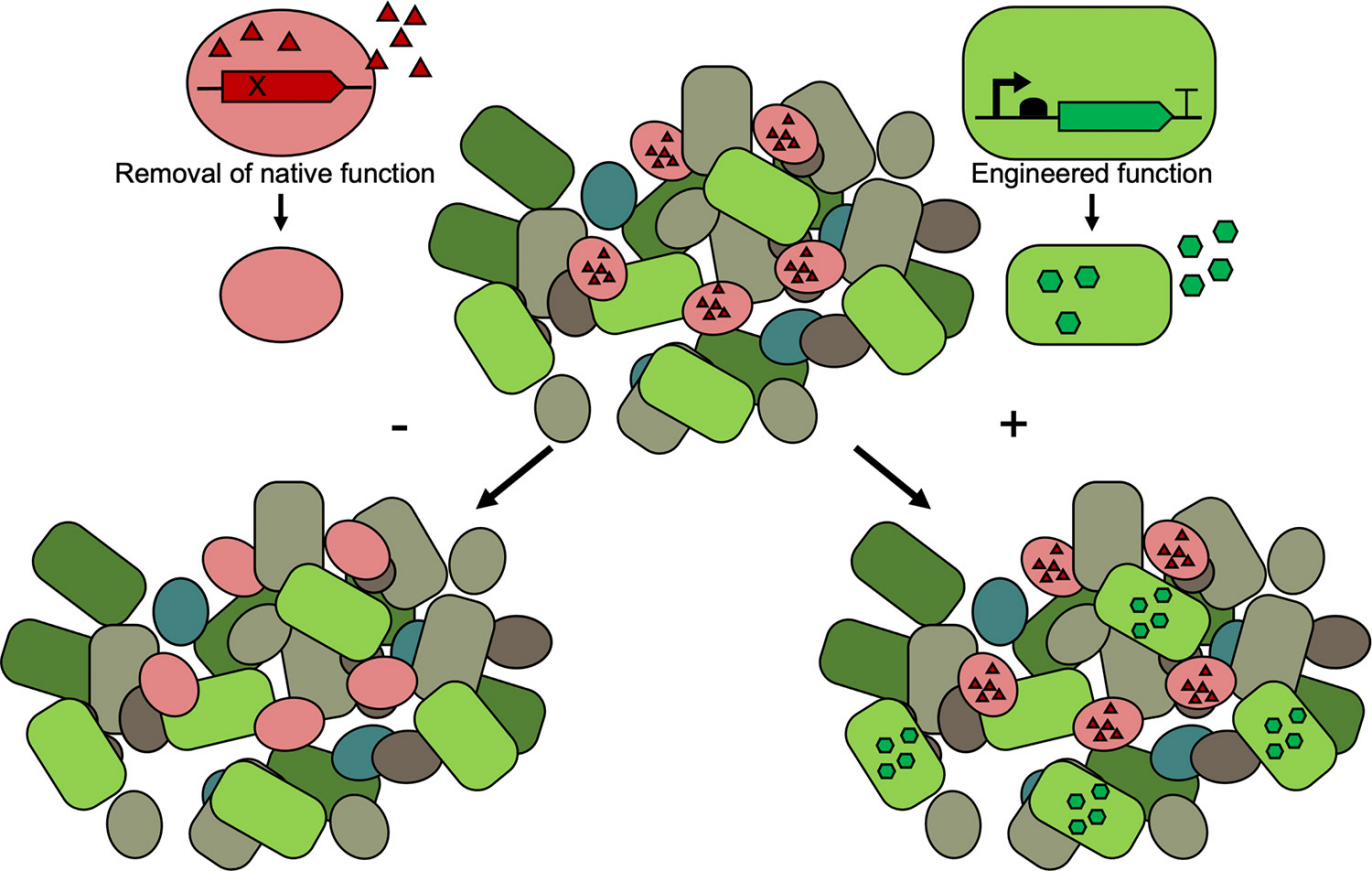
Genetic editing schemes enable functional perturbations while maintaining microbiota composition. Using engineering strategies, the molecular metabolism of endogenous bacteria can be manipulated by adding or removing genes that encode beneficial or deleterious phenotypes.

Molecular tools developed for resident gut bacteria make genetic deletions or additions significantly more efficient. By editing gut microbes, the roles of the microbe and its metabolome in the gut can be studied in a more controlled, physiologic environment. This research approach to editing genetic function *in situ* is essential to mechanistically uncover how a microbe interacts within the microbial community, how it competes in a complex environment, and how its metabolism contributes to host physiology (Fig. 2).

### Loss-of-function strategies.

Genomic tools for both *Bacteroides* and *Clostridium* species have made loss of function deletions or knockdowns in important metabolic genes to assess causal associations between specific microbial metabolites and host functions (77). For example, the deletion of a bile salt hydrolase in B. thetaiotaomicron demonstrated that the modulation of the tauro-β-muricholic bile acid in mouse studies leads to weight loss and changes in the host’s metabolism, transcriptional profile, and immune response pathways (84). A similar approach in Clostridium sporogenes used CRISPR-induced deletions to eliminate pathways that produce microbe-derived molecules and then analyzed how the absence of individual metabolites altered host function. This controlled manipulation of the microbiome revealed a previously unknown immunomodulatory role of microbially derived branched-chain fatty acids (15). Furthermore, genomic deletions in resident gut bacteria have identified incorrect gene annotation assignments that are often heavily reliant on the model E. coli genome (85). Reevaluating and characterizing predicted gene functions can improve the understanding of resident gut microbes and their metabolic pathways. Due to the diversity of the gut microbiota metabolism, targeted knockouts of genes in metabolic pathways are desirable to fully characterize their roles and impacts on host biology.

### Gain-of-function strategies.

With a comprehensive understanding of microbiome niches and refined genome annotations, “nonproducer” microbes can be engineered into “producers” through gain-of-function engineering to evaluate hypotheses in host-microbe interactions. Controllable metabolic pathways can advance our understanding of the microbiome by toggling specific metabolite production and observing the effects in *in vivo* models. In their study, Funabashi et al. identified the genes necessary for the complete production pathways of the bile acids deoxycholic acid (DCA) and lithocholic acid (LCA), two of the most abundant small molecules in the microbiome in Clostridium scindens, an intractable producer. Then, they optimized and transferred the pathway into Clostridium sporogenes, a genetically tractable producer (86). Mobilizing these pathways showcased a method to characterize abundant gut bile acids and demonstrated a genetic proof-of-concept for controlling the microbiome bile acid pool. The ability to modulate bile acids *via* genetic engineering aids in understanding their function. Microbiome-derived metabolites can also be characterized by screening these molecules for particular phenotypes. Campbell et al. screened gut metabolites for the ability to induce regulatory T-cells (T_reg_) and identified the secondary bile acid 3β-hydroxydeoxycholic acid (isoDCA) as a candidate molecule. Multiple *Bacteroides* species (B. thetaiotaomicron, B. fragilis, and B. ovatus) were then engineered to produce isoDCA by transferring hydroxysteroid dehydrogenase genes from the Gram-positive anaerobe [*Ruminococcus*] *gnavus*. When the engineered strains were introduced to a synthetic consortium in mice, isoDCA concentrations in the gut increased and induced host T_reg_ differentiation (Campbell et al. 2020, 14).

Engineering metabolic functions into resident gut bacteria to study small molecule mechanisms are not restricted to bile acid metabolism. Germ-free mice colonized with B. thetaiotaomicron expressing an *R. gnavus*-derived tryptophan decarboxylase effectively converted tryptophan to tryptamine, which increased colonic secretion and accelerated gut transit time (87). This work suggests that engineering bacterial tryptophan metabolism in the microbiome is a promising therapeutic target for diseases associated with slow GI-transit, such as irritable bowel syndrome. Furthermore, specifically inducing native microbial metabolic pathways can highlight their effects on host physiology and uncover novel mechanisms of microbe-host interactions. Engineered gut microbes that can colonize and selectively control the gut environment are effective systems by which to understand the roles of microbe-derived molecules in host physiology and disease.

## DESIGNING RESIDENT GUT BACTERIA FOR USE AS LIVE BIOTHERAPEUTICS

### Wild-type resident gut microbial therapeutics.

The intrinsic features of resident gut microbes make them intriguing candidates for cell-based therapies. Some species may natively produce therapeutic compounds or bolster other therapeutic interventions. Current studies evaluating the applications of wild-type gut bacteria include their roles in immunoprotection, pathogen exclusion, and in supplementing cancer immunotherapy (88–91). For example, some resident gut bacteria, such as those in the families Lachnospiraceae and Oscillospiraceae, naturally produce butyrate, which is thought to be immunoprotective in the mammalian gut (18, 91). Probiotic supplementation with these species holds promise to regulate gut dysbiosis and treat inflammation. Likewise, spore-forming Firmicutes can successfully exclude pathogenic bacteria like C. difficile from the gut and prevent reinfection after antibiotic treatment. This approach has been shown to be effective in reducing C. difficile reinfection compared to a placebo in clinical trials performed by Seres Therapeutics using their SER-109 defined community (89). Lastly, with the expansion of cancer immunotherapy, new roles of the microbiota are being uncovered, particularly in relation to immune checkpoint blockade therapies. An increased relative abundance of specific bacterial species, such as *Bifidobacterium* or *Akkermansia*, has been associated with successful anti-PD-1 treatment (88, 90). While they possess many natural benefits, these intrinsic advantages can be further supplemented by engineering additional functions into certain resident gut bacteria.

### Engineered model bacteria in clinical trials.

Wild-type model microbes such as E. coli Nissle and LAB have likewise shown clinical potential by demonstrating probiotic effects in inflamed and obese mouse models (92, 93). Now, genetically engineered model microbes that produce or metabolize targeted molecules have begun to emerge for therapeutic applications. In a landmark study, the model LAB L. lactis was engineered to secrete interleukin-10 (IL-10), an anti-inflammatory cytokine, to ameliorate colitis in mouse models (12). In a subsequent nonplacebo controlled, phase I trial, Crohn’s disease patients were treated with the IL-10 producing bacteria. Disease activity decreased, and the engineered microbe was determined to be safe (94). This study marked the first genetically engineered microbe to deliver a therapeutic compound in humans. Since then, the field has continued to advance, with more engineered model bacteria entering clinical trials as therapeutics. Recently, an E. coli engineered to metabolize phenylalanine showed promise as a treatment for the metabolic disorder phenylketonuria (11). The clinical trials that followed showed the safety and tolerability of the SYN1618 engineered E. coli and also demonstrated a proof-of-mechanism for the live biotherapeutic by detecting increases in downstream strain-specific metabolites (95).

### Resident gut bacteria engineered to directly produce engineered therapeutics.

Despite their successes, inherent issues remain with using model organisms as therapeutics for certain applications, namely, their poor gut colonization efficiency. Without effective colonization, problems arise with the dosing and delivery of therapeutics to treat any long-term or chronic disorders. Resident gut microbes that can stably colonize the gut and possess intrinsic beneficial properties could subvert many of the challenges facing systems that use model bacteria. For example, *Bacteroides* spp. tend to colonize the gut stably and at high levels and can occupy specific spatial microniches in the gut, such as the intestinal crypts, that might be difficult for model organisms or traditional drugs to access (96). Thus far, *Bacteroides* species, such as B. ovatus, have been successfully engineered to produce potentially beneficial molecules such as interleukin-2 (73), keratinocyte growth factor-2 (74), and transforming growth factor-β1 (75) in order to modulate the host immune system. Despite their functionality *in vitro*, few of these systems have been translated to animal models or to human trials, compared to their model counterparts. Another promising strategy for therapeutic molecule production employs *Bifidobacterium* spp. as an alternative chassis organism. Bifidobacterium bifidum, engineered by Bifidobacteria Expression SysTem (BEST), uses signal peptides to deliver IL-10 to mucosal surfaces in mouse models of colitis (97). Successes with producing therapeutic metabolites in resident gut bacteria suggest promising future applications in other potential beneficial gut microbes, such as butyrate-producing Lachnospiraceae (98) and Akkermansia muciniphila (99) as additional genetic systems income online.

### Metabolic engineering of resident gut bacteria for therapeutics.

Resident gut bacterial chassis can also be engineered to produce beneficial molecules beyond their natural capabilities through metabolic engineering. In one such investigation, butyrate was produced for the first time in a nonproducing commensal bacteria by encoding enzymes for the butyrate biosynthetic pathway into B. thetaiotaomicron (100). While direct incorporation of the pathway did not yield the butyrate at first, optimization using genome-scale metabolic models to tune the microbe’s metabolic network led to successful butyrate synthesis. This approach was not validated *in vivo* but is a promising development for design-based approaches to synthesizing beneficial biological molecules in nonnatural producers. In another example, rooted in evidence gleaned from prior mechanistic studies, B. thetaiotaomicron that were engineered to convert tryptophan to tryptamine increased the concentration of tryptamine in the gut, improved ionic flux across the colonic epithelium, and attenuated weight loss in a DSS colitis mouse model (13). Treating disease by controlling bacterial metabolism *in vivo* can also be achieved by eliminating deleterious microbial functions. For example, a family of tryptophanases in *Bacteroides* produces indole, which is converted to the toxic metabolite indoxyl sulfate that accumulates in the context of chronic kidney disease. The elimination of indole production in B. thetaiotaomicron controlled indoxyl sulfate levels in mouse models, suggesting that renal disease can potentially be managed by targeting the microbiota (101).

### Engineered resident gut bacteria in clinical trials.

For some applications of microbial live biotherapeutics, the engineered microbe will have to engraft in the gut in a safe, consistent, and effective manner. One method to provide a competitive advantage over other microbiota members is to engineer an organism to metabolize a specific carbon source that cannot be degraded by members of the endogenous community. Shepherd et al. incorporated a porphyran utilization operon into B. thetaiotaomicron so that the engineered strain could occupy an orthogonal metabolic niche and engraft into a humanized mouse microbiome when mice were fed a unique carbohydrate that was inaccessible to the native microbiota (102). These methods can be implemented to ensure the engraftment of living therapeutic organisms and to exogenously control their abundance in the microbiome. Novome Biotechnologies has recently paired this colonization strategy in a *Bacteroides* species with an engineered pathway to degrade oxalate for treating enteric hyperoxaluria and progressive kidney damage. The results of phase 1 clinical trials demonstrated that the engineered microbe is safe and can selectively colonize in a dose-dependent manner (103). This represents the first *Bacteroides* live biotherapeutic to reach stage 1 clinical trials and is a promising first step for future resident gut microbe live biotherapeutic applications.

## SAFETY CONCERNS AND BIOCONTAINMENT

Even if proven to be effective treatments, microbial live biotherapeutics have significant safety hurdles to overcome before being widely used and accepted. Both the risk of mutation and the transfer of genetic material are reasons for caution. If they are not contained properly, live biotherapeutic microbes could acquire detrimental functions or unwanted antibiotic resistance and lead to microbiome disruption or potential pathogenicity. Additionally, if resident gut microbes are intended to colonize patients long-term, their abundance in the gut must be tunable. Engineered microbes are being designed with a variety of biocontainment measures to address these issues. A popular containment method is to create auxotrophic microbes that must rely on a continuous supply of a specific nutrient to remain viable. In the first engineered LAB to move into clinical trials, the therapeutic IL-10 gene was inserted in the place of the native thymidylate synthase gene (*thyA*). As a result, when the microbe was deprived of thymidine and thymine, a significant drop in viability was shown (104). However, due to concerns that cross-feeding or gene transfer from other microbiome species will obviate auxotrophy, other biocontainment techniques have been developed. Genetic “kill-switches” that are designed to kill the cell when triggered, can provide patients and clinicians with control over a population. These systems are designed to respond to environmental cues based on the patient’s condition or to prevent spread into the environment once excreted by the host (105, 106). Additional biocontainment strategies focus on preventing the transfer of genetic material, including antibiotic resistance genes, among microbiota members. Multiple-plasmid expression systems and circuit integration into genomes attempt to solve these issues; however, they require longer-term studies to determine escape frequency. Thus far, the results of phase 1 clinical trials using engineered E. coli, LAB, and *Bacteroides* spp. are defining the safety standards for future iterations of live biotherapeutics. Further development of novel biocontainment methods and the combination of existing strategies will increase the long-term stability and general confidence in the safety of engineered microbial therapies.

## FUTURE OUTLOOK

**FIG 3:**
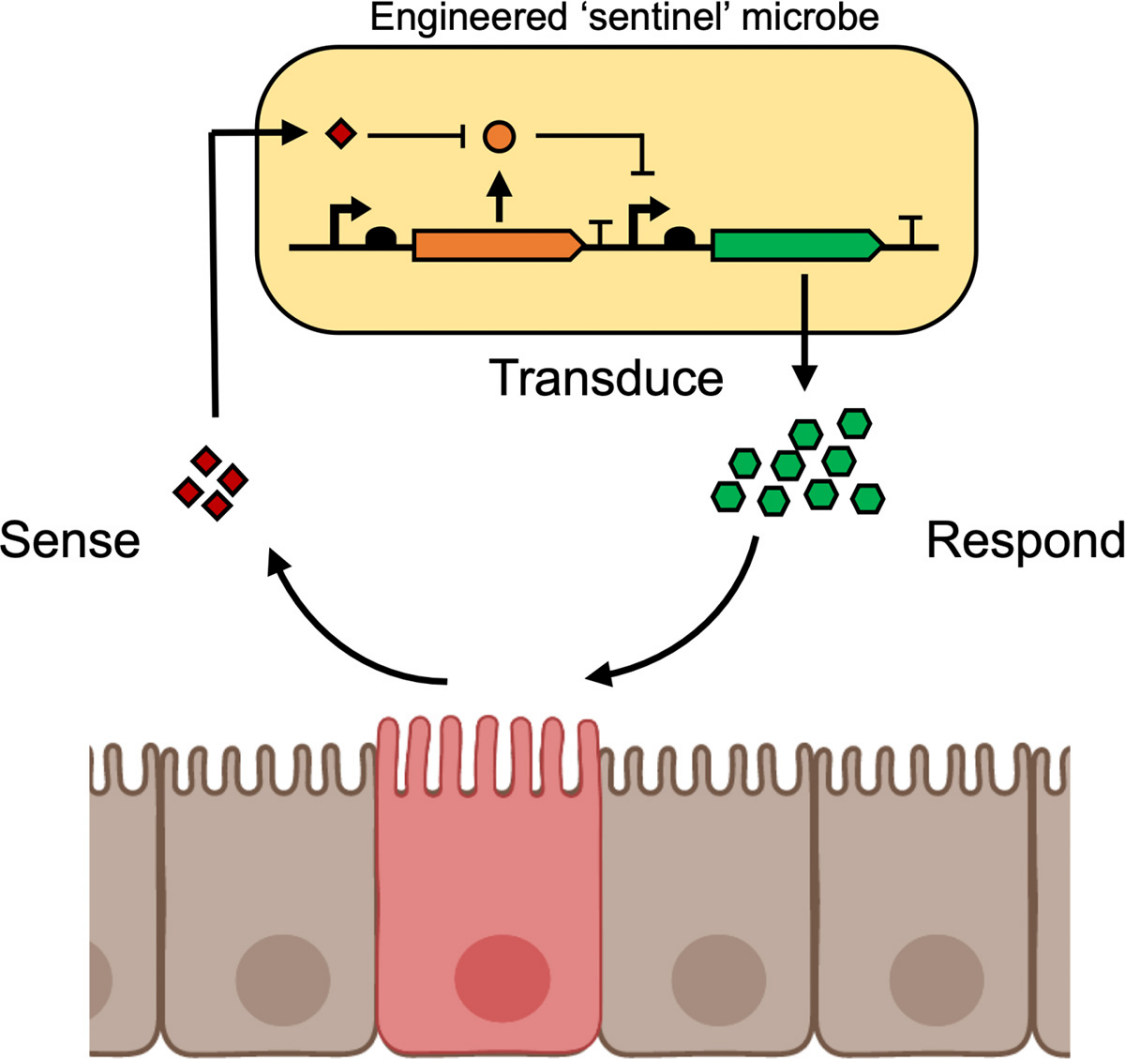
Engineered sentinel microbes as resident therapeutic factories. Microbes acting as sentinels can be engineered to continuously probe the intestinal environment to sense the disease state, transduce these signals to activate engineered pathways, and produce therapeutic compounds in response to the detected disease.

The advancement of genetic tools for resident gut bacteria led to the development of engineered microbes that can probe mechanistic microbiome relationships, manipulate the gut microenvironment, and deploy engineered molecules and functions *in situ*. These microbes, programmed for specific tasks in the gut, promise to benefit human health and have exciting potential as novel biotherapeutics. To continue to build on these successful proof-of-concept studies, researchers must take additional steps to fine-tune novel diagnostic and therapeutic microbial systems as well as to develop sense-and-respond “sentinel” microbes that incorporate these functionalities into a singular deployable biotherapeutic (Fig. 3).

Engineered resident gut microbes are well-suited for gut-related disease diagnostics. Bacteria are naturally adept at sensing their environments, and resident gut bacteria can safely colonize and persist in the gut, making them a viable solution to monitor patients with chronic gut disorders. Initial forays into developing microbial diagnostics led to the creation of E. coli Nissle 1917 (EcN) biosensors that can detect markers of inflammation and intestinal bleeding in animal models (6, 8, 10, 107). Recent work has also implemented model inducible circuits in *Bacteroides* species and has identified and characterized new promoter-based systems by using both computational and functional methods (22, 23, 108). This increase in the diversity of sensor systems broadens the scope of applications available for resident gut bacterial diagnostics, but further advancement is needed for their widespread adoption. Understanding the microbiome’s response to disease when coupled with improved predictive cellular design software, such as Cello, for resident gut bacteria further improves synthetic biology tool and their applications in these chassis (72).

By merging diagnostic sensor systems with therapeutic production pathways, microbial “sentinel cells” can be engineered to sense and respond to disease (Fig. 3). Sentinel cells colonize the host long-term and act as a programmable means of maintaining homeostasis by autonomously activating a therapeutic response when a disease state is detected. Already, first-generation microbial sentinels have shown functionality in model organisms. The live E. coli Nissle 1917 biotherapeutic developed to treat PKU only expresses the necessary enzymes to degrade phenylalanine under anaerobic conditions (11, 95). Recently, E. coli Nissle 1917 was also engineered to detect thiosulfate and release immunomodulatory AvCystatin to treat inflammation *in vivo* (9). Other engineered E. coli targeted to sense and kill various bacterial pathogens have been created to activate in response to specific quorum-sensing molecules or markers of inflammation (11, 12, 109, 110). Additionally, probiotic yeast was engineered to sense and degrade extracellular ATP to treat gut inflammation using a closed-loop genetic circuit. The self-regulated control of enzymatic expression mitigated the fibrotic side effects *in vivo* (111). Complementing these studies, additional genetic manipulation techniques and an expanded repertoire of sensor systems are of critical importance in the further expansion of the applications and compatibility of resident gut bacterial sentinels.

With the inherent advantages of resident gut bacteria and an increasing number of genetic manipulation techniques being developed, resident gut sentinels could also address issues beyond inflammation and specific metabolic disorders. Based on their natural interactions with the host immune system, resident gut sentinel bacteria could be applied to the domain of immunoengineering for applications such as the treatment of autoimmune and inflammatory diseases as well as cancer. Furthermore, the gut-brain axis is another exciting area of research that could benefit from detailed mechanistic studies and sentinel monitoring enabled by gut symbiont engineering (112).

The burgeoning interest in microbiome research necessitates the creation of additional experimental tools for resident gut microbes. When new microbe-disease associations are uncovered, a roadmap should be in place to quickly develop genetic tools to domesticate the relevant resident gut organism and to understand its mechanistic interactions with the microbiota and the host (16, 113). Resident members of the microbiome are an untapped reservoir of potentially beneficial metabolites and other gene products that can be exploited for basic science and therapeutic applications. By characterizing genetic function and domesticating intractable species, therapeutic strategies and sentinel microbes can be designed to incorporate their native beneficial attributes. These natural advantages, including their specialized colonization niche, or the endogenous production of potentially beneficial metabolites could be combined with diverse sensor systems and the production of therapeutic compounds to create potent live biotherapeutics.

The advancements of genomic tools for manipulating gut microbes have spurred the investigation into the mechanistic relationship between the host and the microbiome and have inspired the exploration of live biotherapeutics as viable treatments to mitigate disease. The published examples and proof-of-concept studies outlined in this minireview demonstrate the efficacy and potential for resident gut bacteria as target organisms for microbiome engineering and synthetic biology. The discoveries of additional genetic tools and the novel applications of the use of resident gut bacteria as engineering targets are critical to maintaining the momentum of the field and ensuring that bacterial diagnostics, therapeutics, and sentinel systems achieve their full potential.
